# Stimulation of TLR3 triggers release of lysosomal ATP in astrocytes and epithelial cells that requires TRPML1 channels

**DOI:** 10.1038/s41598-018-23877-3

**Published:** 2018-04-10

**Authors:** Jonathan M. Beckel, Néstor Más Gómez, Wennan Lu, Keith E. Campagno, Bardia Nabet, Farraj Albalawi, Jason C. Lim, Kathleen Boesze-Battaglia, Claire H. Mitchell

**Affiliations:** 1 Department of Anatomy and Cell Biology, University of Pennsylvania, Philadelphia, PA USA; 2 Department of Biochemistry, University of Pennsylvania, Philadelphia, PA USA; 3 Department of Orthodontics, University of Pennsylvania, Philadelphia, PA USA; 4 Department of Ophthalmology, University of Pennsylvania, Philadelphia, PA USA; 5 Department of Physiology, University of Pennsylvania, Philadelphia, PA USA; 6 Department of Pharmacology and Chemical Biology, University of Pittsburgh, Pittsburgh, PA USA

## Abstract

Cross-reactions between innate immunity, lysosomal function, and purinergic pathways may link signaling systems in cellular pathologies. We found activation of toll-like receptor 3 (TLR3) triggers lysosomal ATP release from both astrocytes and retinal pigmented epithelial (RPE) cells. ATP efflux was accompanied by lysosomal acid phosphatase and beta hexosaminidase release. Poly(I:C) alkalinized lysosomes, and lysosomal alkalization with bafilomycin or chloroquine triggered ATP release. Lysosomal rupture with glycyl-L-phenylalanine-2-naphthylamide (GPN) eliminated both ATP and acid phosphatase release. Secretory lysosome marker LAMP3 colocalized with VNUT, while MANT-ATP colocalized with LysoTracker. Unmodified membrane-impermeant 21-nt and “non-targeting” scrambled 21-nt siRNA triggered ATP and acid phosphatase release, while smaller 16-nt RNA was ineffective. Poly(I:C)-dependent ATP release was reduced by TBK-1 block and in TRPML1^−/−^ cells, while TRPML activation with ML-SA1 was sufficient to release both ATP and acid phosphatase. The ability of poly(I:C) to raise cytoplasmic Ca^2+^ was abolished by removing extracellular ATP with apyrase, suggesting ATP release by poly(I:C) increased cellular signaling. Starvation but not rapamycin prevented lysosomal ATP release. In summary, stimulation of TLR3 triggers lysosomal alkalization and release of lysosomal ATP through activation of TRPML1; this links innate immunity to purinergic signaling via lysosomal physiology, and suggests even scrambled siRNA can influence these pathways.

## Introduction

Purinergic signaling involves a complex set of receptors whose activation is controlled by tight spatial and temporal regulation of ATP release. Stimuli such as mechanical stretch^1–3^, chemical stimulation^4^, membrane depolarization^5^, pathogen binding^6^ or hypoxia^7^ can cause the release of ATP from cells. Cellular mechanisms responsible for this release of ATP also vary widely. For example, ATP can be released through large-pore ion channels such as pannexins, calcium homeostasis (CALHM) channels or voltage gated anion channels (VDACs)^8–11^. ATP is released from neurons using traditional synaptic processes, where ATP is stored in and released from vesicles that fuse with the plasma membrane^12–14^. Astrocytes and other cell types also release ATP through vesicular methods^15–18^. Lysosomes are an important source of vesicular ATP release from non-neural cells, with the fusion of lysosomal and plasma membranes leading to ATP exocytosis^19–21^.

The lysosome is emerging as a central organizing hub within the cell, coordinating several pathways including autophagy, energetics and signaling^22^. Lysosomes also participate in defense against invading pathogens through Toll-like receptors (TLRs), leading to phagocytosis of pathogens, maturation of phagosomes by binding with lysosomes, and activation of inflammatory responses^23^. The TLR3 receptor is particularly relevant for the lysosome, with activation triggered by dsRNA from viruses as well as some synthetic RNA molecules^24,25^. While purinergic signaling plays a key role in host-pathogen interactions^26^, the contribution of lysosomal ATP release is unknown. We asked whether stimulation of TLR3s led to release of lysosomal ATP. Our results suggest that stimulation of TLR3 triggers lysosomal alkalization and release of ATP and lysosomal contents from both optic nerve head astrocytes (ONHA) and retinal pigmented epithelial (RPE) cells. Moreover, we demonstrate that 21-nt siRNA, but not 16-nt siRNA, also activates lysosomal ATP release, indicating that commercially available siRNA molecules may trigger this response.

## Results

### TLR3 stimulation triggers release of ATP and lysosomal markers from RPE cells

**Figure 1:**
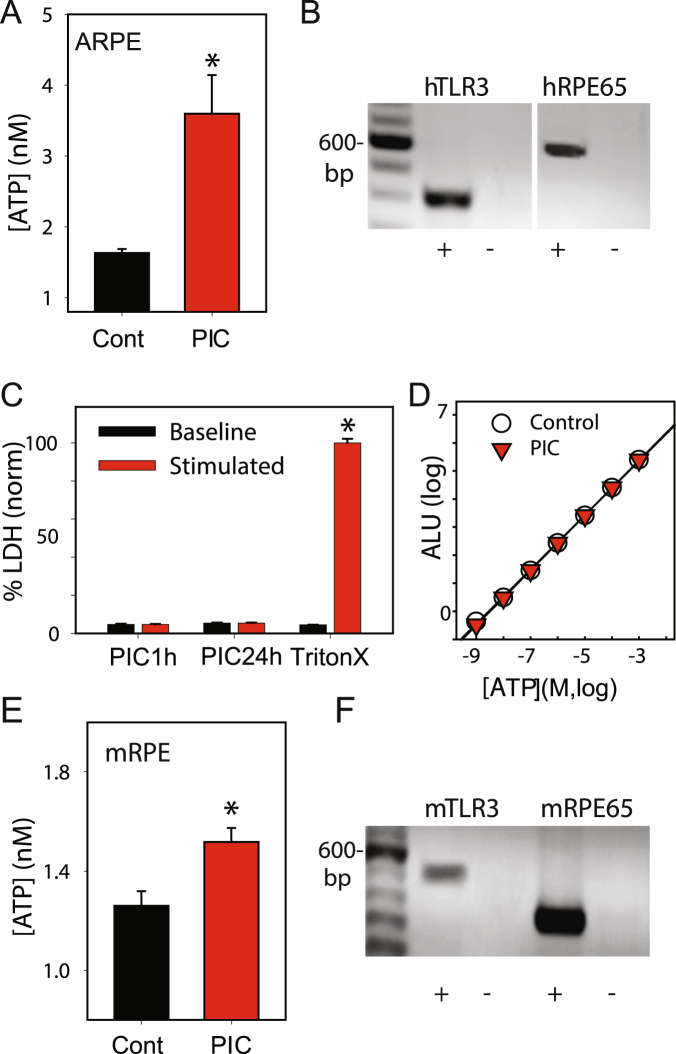
TLR3 stimulation triggers ATP release from RPE cells. (**A**) ATP levels bathing ARPE-19 cells were increased after 20 min exposure to 10 µg/ml poly(I:C) (PIC) (n = 3 trials of 30 wells). (**B**) PCR gel of cultured human ARPE-19 cells showing message for human TLR3 (hTLR3) and RPE-65 (hRPE65); “ + ” with and “−“ without reverse transcriptase. Full gels in Supplemental Figure. (**C**) Poly(I:C) stimulation of ARPE-19 cells for 1 or 24 hrs did not release lactose dehydrogenase (LDH) into the bath but lysing cells with Triton X did; n = 4, p < 0.01. (**D**) ATP standard curve with (red triangles) and without (white circles) 10 µg/ml poly(I:C) show no effect of the drug on the assay; fit with first order regression, n = 3. (**E**) Extracellular levels of ATP bathing mouse RPE cultures (mRPE) after 20 min exposure to 10 µg/ml poly(I:C), n = 18. (**F**) PCR gel demonstrating bands for mouse TLR3 (mTLR3) and marker RPE-65 (mRPE65) in primary cultures of mouse RPE. Results from RPE cells are colored red throughout. Here and throughout the figures, bars represent mean ± SEM.

Initial experiments were performed using the human ARPE-19 cell line. Exposure of these cells to 10 µg/ml of the TLR3 agonist poly(I:C) for 20 min increased extracellular levels of ATP bathing ARPE-19 cells (Fig. 1A). Several controls were performed to determine if this elevation in extracellular ATP was physiological. First, expression of TLR3 and RPE cell marker RPE65 were confirmed using PCR (Fig. 1B; full length gels are included as Supplemental Information Figure S1A and B). Next, levels of lactate dehydrogenase (LDH) did not increase following stimulation of ARPE-19 cells with poly(I:C), with exposure of 1 or 24 hrs (Fig. 1C). This implied the ATP release accompanying poly(I:C) exposure was not due to a generalized cell lysis. Third, the ability of the luciferin/luciferase assay to detect ATP levels was not affected by poly(I:C) (Fig. 1D). Fourth, ATP release was confirmed from mouse RPE cells to ensure the signaling response was also present in primary cells (Fig. 1E, Fig. S1C). Finally, expression of mRNA for TLR3 and cell marker RPE65 were robust (Fig. 1F) in mouse RPE cells.

**Figure 2:**
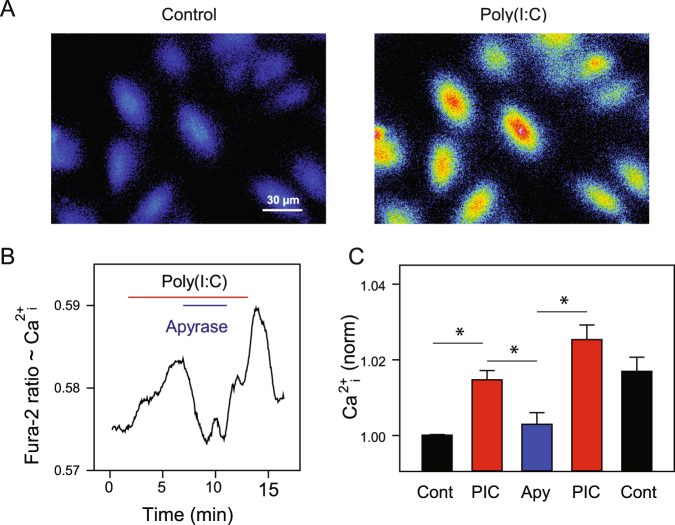
Poly(I:C)-dependent ATP release raises cell calcium. (**A**) Representative images of ARPE-19 cells loaded with Ca^2+^ indicator Fura-2 and excited at 340 nm (em > 540 nm) in control solution (left) and during application of poly(I:C) (30 µg/ml); brighter signal is consistent with higher cytoplasmic Ca^2+^ levels. (**B**) Representative trace from ARPE-19 cell loaded with Fura-2 showing how addition of poly(I:C) (30 µg/ml) elevated the Fura-2 ratio (340/380 nm excitation, >540 nm emission) while the ectoATPase apyrase (10 U/ml) reduced the ratio. (**C**) Summary of responses in cells exposed to poly(I:C) (PIC) and apyrase (Apy) as in A. Ratio values normalized to the mean starting ratio in each cell (n = 11, *p < 0.05). Similar responses found in 4 trials.

To determine whether this released ATP was physiologically relevant, levels of intracellular Ca^2+^ were monitored in ARPE-19 cells with the indicator Fura-2. Poly(I:C) raised the levels of cytoplasmic Ca^2+^ in these cells (Fig. 2A). However, the ectoATPase apyrase produced a significant and reversible reduction in the Ca^2+^ response to poly (IC) (Fig. 2B,C). As apyrase can reduce levels of extracellular ATP^27^, this suggests that released ATP is involved in the Ca^2+^ rise induced by poly(I:C).

**Figure 3:**
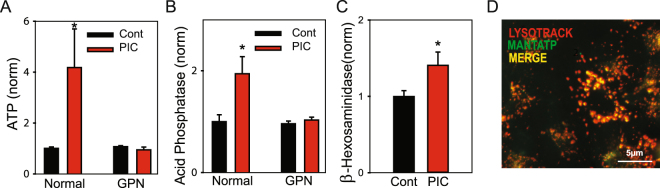
TLR3 and release of lysosomal ATP. Pretreatment of ARPE-19 cells with 200 µM GPN for 60 min to rupture lysosomes prevented the release of ATP (**A** *p = 0.026) and acid phosphatase (**B** *p = 0.041) detected upon exposure to 10 µg/ml poly(I:C) (n = 3–6 for both). (**C**) Exposure to poly(I:C) also increased bath levels of beta-hexosaminidase (*p = 0.040, n = 23). (**D**) Staining of fluorescent ATP marker MANT-ATP (green) merged with LysoTracker (red).

To examine the contribution of lysosomes to the poly(I:C)-mediated response, the effect of glycyl-L-phenylalanine 2-naphthylamide (GPN) was tested; the hydrolysis of GPN by lysosomal enzyme cathepsin C can lead to lysosomal rupture^28^, with treatment dispersing cellular contents^29^. Pre-treatment of RPE cells with GPN abolished the ATP release triggered by poly(I:C) (Fig. 3A). Levels of lysosomal enzyme acid phosphatase were measured to support the lysosomal association of the released ATP. Poly(I:C) led to release of acid phosphatase (Fig. 3B). However, pretreatment of cells with GPN also abolished release of acid phosphatase. Extracellular levels of a second lysosomal enzyme, beta-hexosaminidase, were also elevated after treatment with poly(I:C) (Fig. 3C). Live-cell imaging in ARPE-19 cells showed overlap between fluorescence of N-methylanthraniloyl-ATP (MANT-ATP) and the lysosomal marker LysoTracker Red (Fig. 3D), suggesting concentrated stores of ATP were present in lysosomes.

**Figure 4:**
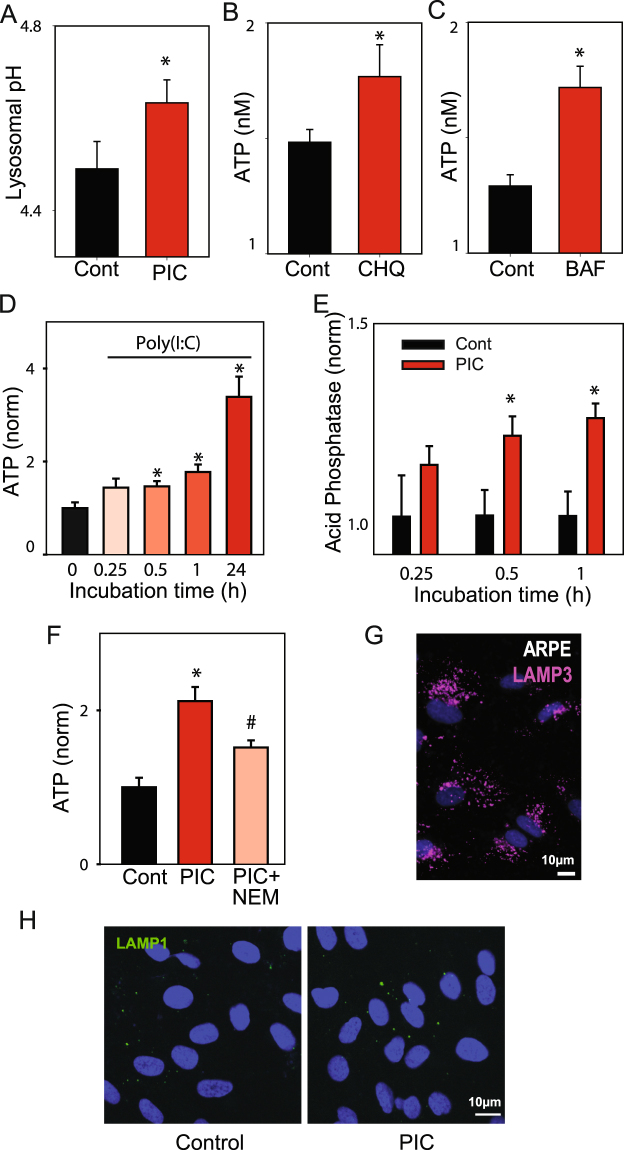
Lysosomal alkalinization and vesicular release in RPE cells. (**A**) Exposure to poly(I:C) produced a small but significant elevation of lysosomal pH in ARPE-19 cells (n = 4 trials with 20 wells). Raising lysosomal pH with either 10 µM chloroquine (**B** CHQ, n = 5 trials of 30 wells) or 8 µM bafilomycin A1 (**C** BAF, 3 trials with 30 wells) for 20 min led to ATP release. The time-course of ATP release (**D**) paralleled that of acid phosphatase release (**E**) following exposure to 10 µg/ml poly (I:C). Levels are normalized to control ATP/acid phosphatase levels for each trial (3 trials with 20+wells). *p < 0.05 vs. control, ^#^p < 0.05 vs. poly(I:C) alone. (**F**) ATP release from cells exposed to 10 µg/ml poly(I:C) was reduced by 1 mM N-ethylmaleimide (NEM, n > 30). (**G**) ARPE-19 cells stained for secretory lysosome marker Lamp3 (CD63, magenta) and DAPI (blue). (**H**) Exposure of cells to poly(I:C) for 30 min increased staining for the luminal epitope of LAMP1 (green) on the cell surface.

Additional experiments examined the effect of poly(I:C) on lysosomes. Poly(I:C) led to a small but significant rise in lysosomal pH (Fig. 4A). The corollary was also true; chloroquine (Fig. 4B) or bafilomycin A1 (Fig. 4C) led to a release of ATP into the bath similar to that found with poly(I:C). As both drugs can alkalinize lysosomes in these cells^30^, these results suggest alkalinization is associated with lysosomal release. The time course of both ATP (Fig. 4D) and lysosomal enzyme acid phosphatase release (Fig. 4E) were parallel, with extracellular levels increasing with exposure time. The vesicular fusion inhibitor N-ethylmaleimide (NEM) reduced ATP release in RPE cells following exposure to poly(I:C) (Fig. 4F). As NEM blocked lysosomal release from monocytes^20^, supporting vesicular fusion in the ATP release triggered by poly(I:C) here, and ARPE-19 cells expressed abundant levels of secretory lysosome marker Lamp3 (CD63)^31^ in vesicle-like structures (Fig. 4G)^32^. Exposure of cells to poly(I:C) also led to a modest increase in staining for the luminal epitope of lysosomal protein LAMP1 as compared to controls (Fig. 4H).

### TLR3 stimulation triggers rapid release of ATP and lysosomal enzymes from astrocytes

**Figure 5:**
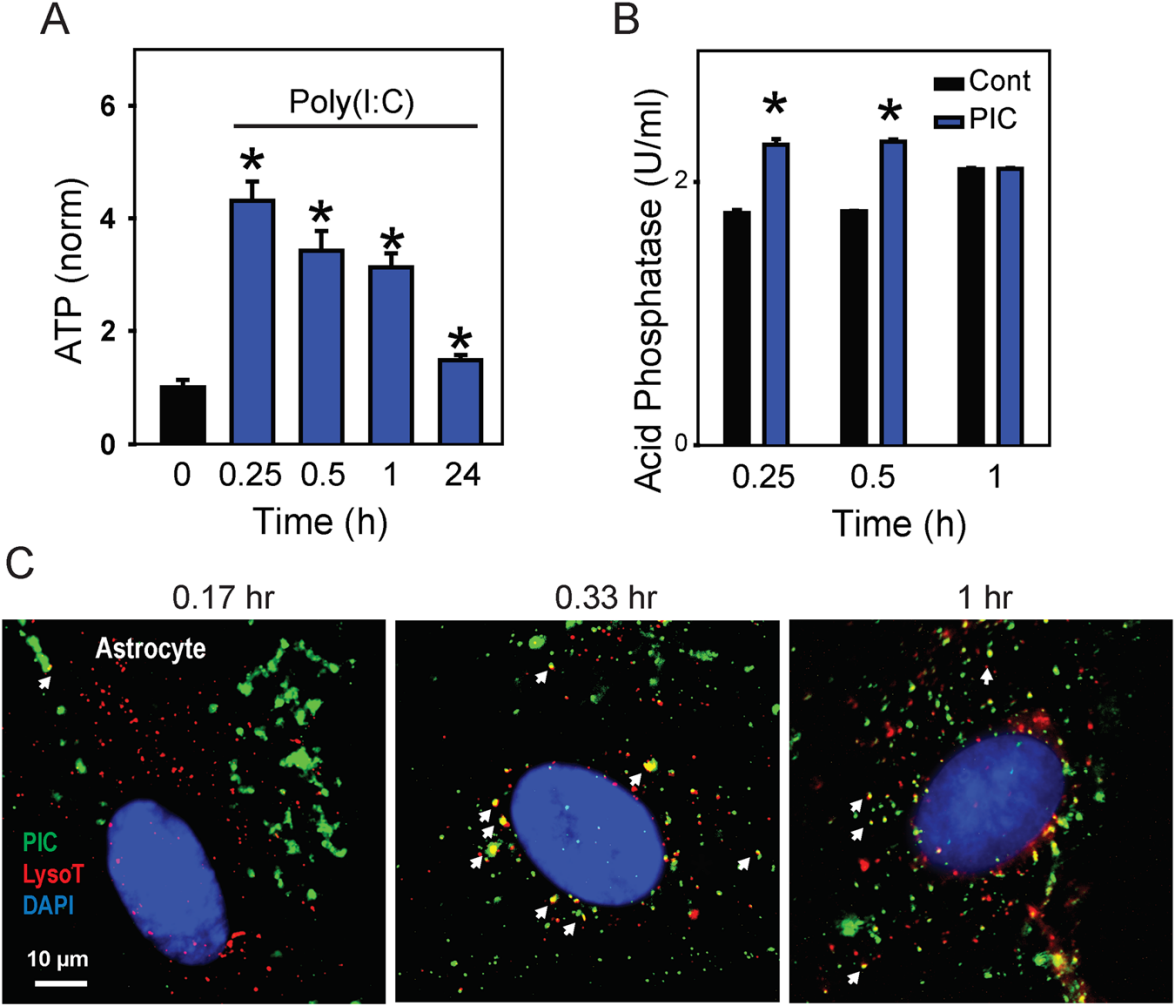
TLR3 stimulation triggers ATP release from rat optic nerve head astrocytes. (**A**) The time-dependence of ATP release from astrocytes, with levels recorded at the indicated time after addition of 10 µg/ml poly(I:C); ATP concentration was normalized to mean control for each trial, n ≥ 30. Results from astrocytes are colored blue throughout. (**B**) The release of acid phosphatase in response to poly(I:C) (PIC) followed a similar rapid time course to the ATP release. *p < 0.05 vs. control, n = 6–10. (**C**) Colocalization of fluorescent poly(I:C) (green) and LysoTracker Red at 10, 20 and 60 min after first exposure to fluorescent poly(I:C). Overlap is indicated by white arrows.

To determine whether the efflux was a generalized response to TLR3 stimulation, the ability of poly(I:C) to trigger release was also examined in astrocytes. The astrocytic identity the of primary cultures isolated from the rat optic nerve head was confirmed by the presence of astrocyte marker GFAP with PCR (Fig. S1D) and by GFAP-positive staining using immunohistochemistry (Fig. S1E). Addition of 10 µg/ml poly(I:C) led to a rise in extracellular ATP surrounding astrocytes (Fig. 5A). In contrast to the epithelial cells, the ATP release peaked at 15 min and levels declined with prolonged exposure to poly(I:C). Lack of LDH release suggested that the ATP release was not due to cell lysis (Fig. S1F). The time course of acid phosphatase release after poly(I:C) addition paralleled that of the ATP release in astrocytes and was also distinct from that seen in RPE cells (Fig. 5B), further supporting a physiological release.

The time course of poly(I:C) delivery to the lysosomes was confirmed microscopically using fluorescently-tagged poly(I:C) (Fig. 5C). After only 10 min exposure, most of the fluorescent poly(I:C) did not colocalize with lysosomes and was present in larger amorphous clumps, consistent with its presence on the exterior of the membrane. However, after 20 min, overlap between the fluorescent poly(I:C) and LysoTracker Red was clearly evident, consistent with delivery of the poly(I:C) to the lysosomes by the point when release of ATP and acid phosphatase was detected. Overlap was reduced but remained in cells examined 60 min after the initial presentation of fluorescent poly(I:C).

**Figure 6:**
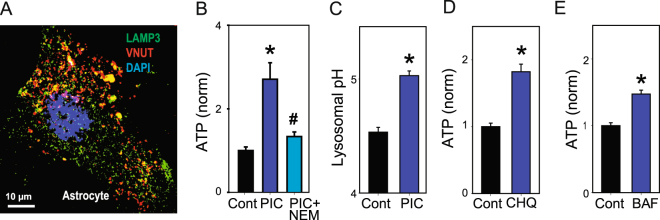
Lysosomal alkalinization and vesicular release in astrocytes. (**A**) Co-staining for the vesicular nucleotide uptake transporter (VNUT, red) and LAMP3 (green) in astrocytes, showing overlap in some locations. (**B**) The ATP release triggered by poly(I:C) was inhibited by 1 mM NEM (n = 20). (**C**) Poly(I:C)(10 µg/ml) raised lysosomal pH in astrocytes (n ≥ 30). Alkalinization of astrocyte lysosomes with chloroquine (**D)** CHQ, 10 µM, n ≥ 30) and bafilomycin A1 (**E**) BAF, 10 ng/ml, n = 30) increased concentration of ATP in the bath. *p < 0.05 vs. control, ^#^p < 0.05 vs. poly(I:C) alone.

Lysosomal involvement was also suggested in the TLR3-mediated ATP release from astrocytes. To determine whether ATP was stored in astrocytic lysosomes, staining patterns of the vesicular nucleotide uptake transporter (VNUT) and secretory lysosome marker LAMP3 were examined. VNUT was present in discrete clusters that overlapped with LAMP3 staining in places (Fig. 6A). The release of ATP triggered by poly(I:C) was prevented by NEM (Fig. 6B); the pannexin blocker probenecid did not affect the ATP release triggered by poly(I:C) in spite of its ability to prevent stretch-mediated ATP release from these cells (Fig. S1G). Together these results are consistent with ATP storage in secretory lysosomes and release through vesicular fusion, and supports the concurrent release of lysosomal acid phosphatase and ATP in response to TLR3 stimulation. To examine whether lysosomal alkalization contributed to ATP release in astrocytes, the lysosomal pH was determined. Poly(I:C) alkalinized lysosomes in astrocytes (Fig. 6C). Lysosomal alkalinization by chloroquine (Fig. 6D) or bafilomycin A (Fig. 6E) was sufficient to induce ATP secretion from astrocytes, as in RPE cells.

### Lysosomal ATP release a general response to TLR3 agonists including scrambled siRNA

**Figure 7:**
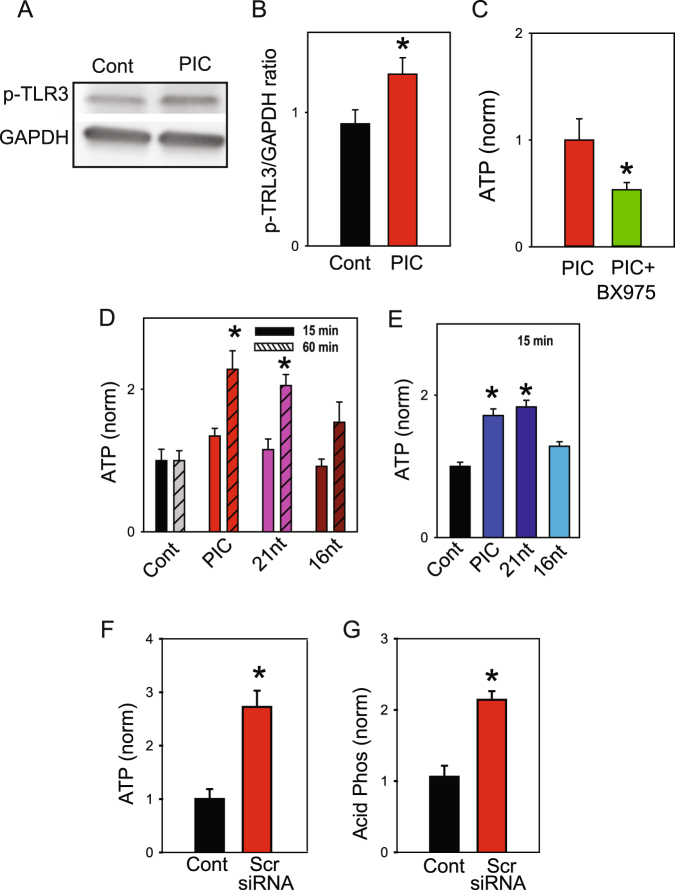
TLR3 stimulation. (**A**) Stimulation of ARPE-19 cells with 10 µg/ml poly(I:C) (PIC) for 5 min increased levels of phosphor-TLR-3 Tyr759 receptor over control (Cont). Representative immunoblot, showing increased phosphorylated band after only 5 min. Bands are ~30 kD p-TLR3, ~37 kD GAPDH. Full blot in Supplemental Fig. 1. (**B**) Densitometry quantification of increased pTLR3 band size normalized to GAPDH, (n = 6, p < 0.05). (**C**) Release of ATP triggered after 30 min in 20 µg/ml poly(I:C) was blocked in astrocytes that were incubated in 10 µM BX-975 for 6 hrs. (**D**) ATP release from ARPE-19 cells was stimulated by 21-nt *Luc* siRNA (5 µg/ml) but not 16-nt *Luc* siRNA (5 µg/ml), with an increase after 60 min exposure but not 15 min. The time course of ATP release induced by the 21-nt *Luc* siRNA paralleled that of poly(I:C) (10 µg/ml). (**E**) The ATP release by 21-nt *Luc* siRNA but not 16-nt *Luc* siRNA was also observed in astrocytes (shown in blue, only 15 min tested). (**F**) Exposure to commercially available non-targeting scrambled siRNA (Scr siRNA, 5 µg/ml) led to a release of ATP (n = 20, p < 0.001) and acid phosphatase (**G)** n = 4, p < 0.001) from ARPE-19 cells at 30 min.

While the preceding data indicate TLR3 agonist poly(I:C) leads to the release of lysosomal ATP, the role of TLR3 was examined. Levels of phosphorylated TLR3 (Tyr759) were increased by poly(I:C) after only 5 min exposure to poly(I:C), implicating receptor phosphorylation as an early step in the response (Fig. 7A,B, Fig. S1H). The release of ATP triggered by poly(I:C) was reduced by inhibiting TANK-binding kinase 1 (TBK-1) with BX-795 (Fig. 7C). As TBK-1 is activated downstream from TRAM-TRIF^33^, this strengthens the role of TLR3 and a non-MyD88 pathway in the ATP release triggered by poly (I:C).

Several studies indicate nonspecific siRNA constructs can stimulate TLR3s and trigger responses, with the length of the nucleotide strand affecting receptor dimerization and the cellular response^25,34,35^. To determine whether these non-specific nucleotides induced release of lysosomal enzymes and ATP in a similar size-dependent manner, we synthesized these same 21-nt *Luc* siRNA and 16-nt *Luc* siRNA constructs against the firefly luciferase gene (see Table 1), and tested their effect on the release of ATP. The 21-nt *Luc* siRNA triggered an ATP efflux from ARPE-19 cells comparable to the release with poly(I:C), producing a robust release detected after 60 but not 15 min (Fig. 7C). In contrast, the 16-nt *Luc* siRNA did not induce a significant release. The non-specific siRNA constructs had an analogous effect on astrocytes, with the 21-nt *Luc* siRNA, but not the 16-nt *Luc* siRNA, leading to ATP release at 15 min (Fig. 7D).

**Table 1:** Primers used to demonstrate gene expression or sequences to activate TLR3.

Gene	Forward primer (5′ to 3′)	Reverse primer (5′ to 3′)	Size (bp)
rTLR3	GATTGGCAAGTTATTCGTC	GCGGAGGCTGTTGTAGG	209
rGFAP	GCCGCTCCTATGCCTCCTCCGA	TCCAGCGACTCAACCTTCCTCT	547
hTRL3	CGGGCCAGCTTTCAGGAACCTG	GGCATGAATTATATATGCTGC	400
hRPE65	GTTTCTGATTGTGGATCTC	GGGATGTTAATCTCCACTTC	603
mTLR3	CAAATCCACTTAAAGAGTTCTCCCCG	CACCAATCCCGTGAAGGTATTGCTT	521
mRPE65	ATGACTGAGAAGAGGATTGTC	CTGCTTTCAGTGGAGGGATC	367
21-nt-siRNA	UAAGGCUAUGAAGAGAUACdTdT	From^25^	21
16-nt-siRNA	UAAGGCUAUGAAGAdTdT	From^25^	16

Commercially available “non-targeting” siRNA was examined for its ability to trigger lysosomal release. This scrambled negative control siRNA is reported to have no similarity to human mouse or rat transcript sequences, and while the sequence is proprietary the manufacturer confirmed the product was at least 21 nucleotides in length. This scrambled siRNA led to an release of both ATP (Fig. 7E) and acid phosphatase (Fig. 7F). This suggests release of lysosomal ATP as a general response of cells to a variety of TLR3 agonists including commercially available non-specific siRNA. Interestingly, addition of lipopolysaccharide (LPS, 1 µg/ml 0111:B4) for 1 hr also increased the ATP release from ARPE-19 cells (Fig. S1I).

**Figure 8:**
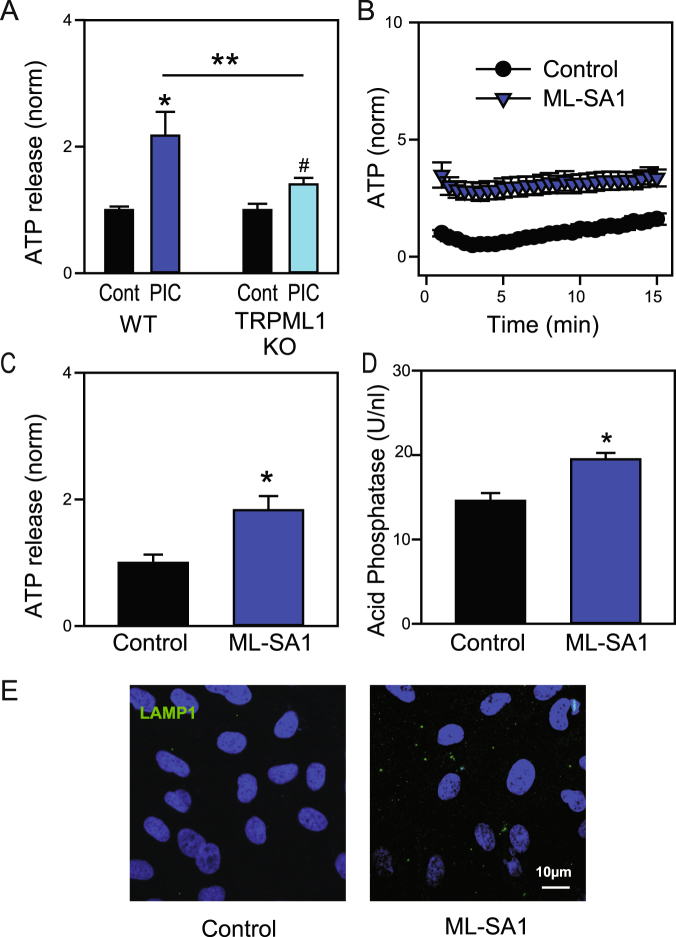
Role of TRPML1. (**A**) The ATP release triggered by PIC in mouse astrocytes was significantly reduced in astrocytes from the TRPML1 knockout mice. (n = 15, *p < 0.01; **p < 0.05 paired Student’s t-tests). (**B**) Levels of ATP released into the bath after addition of 20 µM ML-SA1 to astrocytes (n = 14, 13). (**C**) Mean levels of ATP released into the bath surrounding astrocytes 15 min after addition of ML-SA1 (n = 14, 13, p < 0.001). (**D**) Mean levels of acid phosphatase in bath surrounding astrocytes 60 min after addition of 20 µM ML-SA1 (n = 8, p = 0.001). (**E**) Exposure of ARPE-19 cells to 20 µM ML-SA1 for 30 min increased staining for the luminal epitope of LAMP1 on the cell surface.

### Role for TRPML1

TRPML1 is a lysosomal cation channel implicated in fusion of lysosomal membranes with the plasma membrane in exocytosis^36^. Astrocytes from TRPML1 KO mice were examined to determine whether the channel contributed to the release of ATP in response to TLR3 stimulation. The release of ATP from the TRPML1 KO cells was significantly reduced when compared to wild-type cells (Fig. 8A), suggesting a lysosomal ATP release required TRPML1. We have recently demonstrated that lysosomal signaling is diminished in cells from the TRPML1 KO mouse^29^. To determine whether activity of TRPML1 was sufficient to trigger lysosomal ATP release, the small molecule ML-SA1 was examined as it activates TRPML channels, and can trigger the release of lysosomal Ca^2+^, a key step in fusion of lysosomes with other membranes^37–39^. Exposure of astrocytes to 20 µM ML-SA1 led to a sustained increased in the level of ATP in the extracellular space (Fig. 8B,C). ML-SA1 application also led to a significant increase in acid phosphatase into the bath (Fig. 8D). Finally, ML-SA1 increased levels of the luminal epitope of LAMP1 on the surface, consistent with fusion of the lysosomal and plasma membrane. Together, these findings suggest a role for the TRPML1 channel in the lysosomal ATP release triggered by TLR3.

### Autophagy and lysosomal release

**Figure 9:**
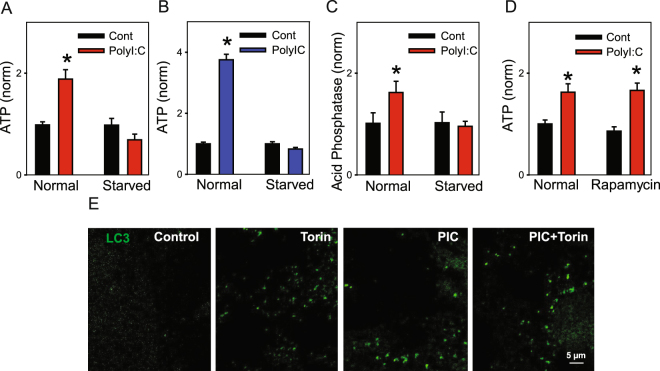
Induction of autophagy and release of lysosomal ATP. (**A**) Starvation of ARPE-19 cells prevented the release of ATP by 30 min poly(I:C) (10 µg/ml) ATP levels were normalized to the mean control for each plate (n ≥ 30), *p < 0.001 vs control. (**B**) Starvation of astrocytes (blue) prevented the release of ATP accompanying 15 min exposure to 10 µg/ml poly(I:C). *p < 0.001 vs control. (**C**) Cell starvation prevented acid phosphatase release in ARPE-19 cells treated with poly(I:C) for 30 min. *p = 0.01 vs control. (**D**) Exposure of ARPE-19 cells to 1 µM rapamycin for 24 hrs did not prevent the release of ATP triggered by poly(I:C). (n = 30, *p < 0.0035) (**E**) RPE cells isolated from LC3-GFP mice show clusters of LC3 with Torin1. Staining was sometimes observed with poly(I:C) but this was not always consistent. Control – untreated, Torin – 3 hrs 1 µM Torin1, PIC – 3 hrs 10 µg/ml poly(I:C), PIC + Torin - combined for 3 hrs.

Conditions known to manipulate mTOR were examined for their impact on lysosomal ATP exocytosis as stimulation of TLR3 activates AKT which in turn activates the mammalian target of rapamycin (mTOR1)^40,41^. First, the ability of poly(I:C) to trigger ATP release was compared in cells grown in standard conditions with 10% serum, or in cells starved of serum for 24 hrs. Cell starvation completely prevented the release of ATP in ARPE-19 cells (Fig. 9A). The poly(I:C) triggered release of ATP was also eliminated in starved astrocytes (Fig. 9B). To determine whether this reflected a specific reaction of starvation on cellular energetics, levels of acid phosphatase were also measured. Cell starvation also eliminated the rise in acid phosphatase induced by TLR3 (Fig. 9C). In contrast, a 24 hr treatment with rapamycin had no effect on ATP release (Fig. 9D) triggered by poly(I:C). RPE cells from LC3-GFP mice were examined to further investigate the relationship between poly(I:C) and autophagy using the more selective mTOR inhibitor Torin 1 (Fig. 9E). In control conditions, fluorescence was disperse and at the limits of resolution. Treatment of cells with poly(I:C) for 3 hrs increased particulate fluorescence, with typical cells displaying multiple LC3 puncta indicative of autophagosomes^42^. Cells exposed to Torin 1 showed particulate fluorescence equal to or often greater that that observed with poly(I:C). Cells treated with both poly(I:C) and Torin 1 were the same as those treated with Torin1 alone. This general pattern was observed in three independent trials, suggesting poly(I:C) induced aggregation of LC3 and did not interfere with induction by Torin1.

## Discussion

This study suggests that stimulation of TLR3 leads to the efflux of lysosomal enzymes and ATP. The data from rat optic nerve head astrocytes and from retinal pigmented epithelial cells of human and mouse origin suggests this link between TLR3 and lysosomal ATP release is a widespread response across species and in cell types traditionally considered non-immune. The ability of several non-specific RNA constructs including poly(I:C), impermeable 21-nt *Luc* siRNA, and non-targeting scrambled siRNA to induce lysosomal ATP release suggest this response may be contribute to events under both experimental and endogenous conditions. The ability of released ATP to raise cell calcium suggests this response may also have physiologic relevance for general cell signaling.

Several observations suggested that the release of ATP triggered by TLR3 receptor activation was lysosomal. First, lysosomal storage of ATP was supported by the colocalization of VNUT with LAMP3 in fixed cells, and by LysoTracker with MANT-ATP using live cells. Next, the release of lysosomal enzymes acid phosphatase and β hexosaminidase following treatment with poly(I:C) support lysosomal release; the parallel time courses of ATP and acid phosphatase release in different cell types provide additional support. The elimination of both ATP and acid phosphatase release following treatment with GPN also implicates lysosomes, as we recently showed GPN dramatically reduced the number of functional lysosomes in RPE cells^29^. Finally, that ability of NEM to inhibit the ATP release from both the epithelial cells and astrocytes resembles its ability is to block lysosomal ATP release from monocytes^20^. The soluble NEM-sensitive factor attachment protein receptor (SNARE) factors Syntaxin 7 and VAMP-7 are required for lysosomal fusion in macrophages^43^, and release of both ATP and lysosomal enzyme cathepsin B were dependent upon the SNARE VAMP7 in glial cells^44^. Together, these observations strongly implicate the lysosomes in the poly(I:C)-dependent release.

The signaling pathways linking TLR3 to lysosomal alkalization and release of ATP are predicted to involve mTOR. Stimulation of TLR3 leads to TRIF-dependent phosphorylation of AKT^40^, while AKT enables RHEB to retain mTOR at the lysosomal membrane^45^. The ability of TBK1 inhibition with BX-795 to prevent the ATP release induced by poly(I:C) supports involvement of this pathway. Although mTOR can regulate lysosomal activity indirectly through TFEB^46^, mTOR may also regulate vHATPase, and thus lysosomal pH, directly^47^. In RPE cells, the crystallin CRYBA1 acidifies lysosomes through direct contact with vHATPase ATP6V0A1 subunit through a mechanism involving mTOR^48^. Bafilomycin A1 and chloroquine alkalinize the lysosome through different mechanisms, so their common actions on ATP release, combined with the lysosomal alkalization caused by poly(I:C), suggest alkalization may itself contribute to lysosomal exocytosis as found elsewhere^49^. The lysosomal alkalization was more robust in astrocytes than in RPE cells, and the rise in lysosomal markers in the extracellular space occurred more rapidly in these cells, consistent with alkalization leading to this release. Whether this pathway contributes to the ATP release by poly(I:C) remains to be determined directly.

The lysosomal cation channel TRPML1 may play a key role in the lysosomal ATP release triggered by TLR3. Lysosomal alkalization can trigger efflux of Ca^2+^ from lysosomal lumen to the cytoplasm through theTRPML1 channel^50^, and data above suggest a role for TRPML1 in the response to TLR3. We have recently demonstrated that pretreatment of RPE cells with GPN prevents the release of lysosomal Ca^2+^ by ML-SA1^29^, consistent with the ability of GPN to prevent the poly(I:C)-dependent release in the present study. This elevated cytoplasmic Ca^2+^ can in turn activate a synaptotagmin VII-associated fusion of lysosomal and plasma membranes, resulting in lysosomal exocytosis^36,51^. This provides an outline linking TLR3 stimulation with lysosomal alkalization, release of lysosomal Ca^2+^ through TRPML1, and subsequent fusion of the lysosomal membrane with the plasma membrane resulting in ATP release, although this requires further confirmation.

TLR3s are present on both plasma membranes and lysosomal/endosomal membranes and respond to extracellular and intracellular signals^52,53^. The ability of unmodified siRNA to trigger the lysosomal ATP efflux implies the involvement of plasma membrane TLR3s, as the 21-nt *Luc* sequence used here is unable to cross the plasma membrane of epithelial or endothelial cells unless conjugated to cholesterol^25,34^. Likewise, the unmodified scrambled siRNA is not expected to be internalized until after receptor binding. The TLRs undergo dimerization and phosphorylation upon agonist binding, with the 21-nt Luc siRNA, but not the 16-nt Luc siRNA, sequences used here shown to induce TLR3 dimerization^25^. Our study demonstrated TLR3 was phosphorylated at Tyr759 within 5 min, analogous to the early stage phosphorylation at 10 min endothelial cells^34^. The absence of specific antagonists to TLR3 also make these parallels with 16-nt and 21-nt Luc siRNA useful at implicating the TLR3 receptor. While the lysosomal ATP efflux triggered by non-targeting scrambled siRNA is consistent with a TLR3 response, it suggests that extra caution should be given to the interpretation of siRNA experiments related to lysosomes and autophagy; increased extracellular ATP and lysosomal enzymes, combined with a decrease in lysosomes, may well accompany even negative controls. This also calls into question the usefulness of treatment paradigms for pathology that utilize siRNA gene therapy, as expression of dsRNA molecules could activate TLRs and elicit inflammatory responses. Shifting to shorter siRNA constructs of 16 base pairs is predicted to avoid this side effect and should be considered, especially in experiments involving autophagy-related questions.

The endogenous ligand for TLR3 in optic nerve head astrocytes and RPE cells remains open to speculation. TLR3 is classically activated by viral dsRNA, and there is some evidence of viral inflammation as a cause for secondary glaucoma^54^. Many cell types have been shown to release nucleic acids in the form of exosomes^55,56^, and while no evidence yet exists that suggests exosome-derived RNA can activate TLR3, other TLRs such as TLR7/8 can be activated by ssRNA^57^. Thus, the possibility exists that TLR signaling in optic nerve head astrocytes or RPE cells could be triggered by endogenous signals and not solely by viral infection. The novel link between the innate immune system, lysosomal efflux and ATP release described here will likely impact multiple systems. P2Y receptors are present on RPE cells and astrocytes, and can be associated with cytokine secretion and fluid regulation^58–60^. Both cell types contain P2X7 receptors, which have been linked to inflammasome activation^61^. We propose the induction of lysosomal ATP efflux by TLR3 lies at the crossroads of several innate immune pathways.

## Methods

### Mouse RPE cell culture

All animal procedures were approved by the University of Pennsylvania Institutional Animal Care and Use Committee (IACUC) protocols #803584 and #804588 in compliance with the Public Health Service Policy on Humane Care and Use of Laboratory Animals. Mice were reared at 5–15 lux and sacrificed with CO_2_. Eyes from mice of both sexes were isolated and processed as described^61^. C57BL/6 J and LC3-GFP mice were obtained from Jackson Laboratories (Bar Harbor, ME). To obtain RPE cells, intact eyes from either gender were incubated in 2% dispase and 0.4 mg/ml collagenase IV for 45 min, rinsed and incubated in growth medium for 20 min (containing Dulbecco’s modified Eagle medium (DMEM) with 1× minimum essential medium + non-essential amino acids, 3 mM L-glutamine, 100 U/ml penicillin, 100 μg/ml streptomycin and 2.5 mg/ml Fungizone and/or 50 μg/ml gentamicin, plus 10% fetal bovine serum (FBS); all Invitrogen Corp). In some experiments, the anterior segments and retinas were removed and the eyecup was rinsed with Versene (Dow Chemical) and incubated in 0.25% trypsin for 45 min. Cells from 4 eyes were pooled and sheets of RPE cells were separated from the choroid and triturated to single cells.

### Astrocyte culture

Primary cultures were grown from Long-Evans rat optic nerve head astrocytes as described^1^. In brief, rat pups of either gender were sacrificed by P5, and the optic nerve head digested for 1–2 hours with 0.25% trypsin. After washing, cells were grown in DMEM/F12, 10% FBS, 1% penicillin/streptomycin, and 50 ng/ml epidermal growth factor (EGF). Cells were used up to passage 5. Cultures were found to contain >99% astrocytes, as defined by glial fibrillary acidic protein (GFAP) immunofluorescence staining. Mouse optic nerve head astrocytes tissue was collected from 3-month-old C57BL/6 J and TRPML1^−/−^ mice of both genders using a similar protocol but with only 35 min of trypsin incubation. TRPML1^−/−^ mice were obtained from Susan Slaugerhaupt of Harvard University^62^; mice were genotyped for TRPML1 as described^62^.

### ARPE-19 cell

ARPE-19 cells (American Tissue Type Collection, Manassas, VA) were grown to confluence in 25 cm^2^ primary culture flasks in a 1:1 mixture of DMEM/F12 medium with 3 mM L-Glutamine, 100 µg/ml streptomycin and 2.5 mg/ml Fungizone and/or 50 µg/ml gentamicin and 10% fetal bovine serum (all Invitrogen Corp) except where indicated. Cells were incubated at 37 °C in 5% CO_2_ and passaged weekly. Cells were typically grown for 2+ weeks before experiments. Confirmation of RPE cell marker RPE65 was performed.

### Polymerase chain reaction

Total RNA was extracted from rat optic nerve head astrocytes, ARPE-19 cells and mouse RPE/choroid complex using the TRIzol reagent (Invitrogen) as described^1,63–65^. Random hexamers were used to reverse transcribe 1 μg of total RNA at 37 °C for 120 min using High-Capacity cDNA Reverse Transcription Kit. Primers were designed in house using Primer 3 software (http://primer3.wi.mit.edu/) (see Table S1). PCR products were visualized on a 2.0% agarose gel in TAE buffer using ethidium bromide to stain DNA.

### ATP measurement

ATP was measured as reported before with minor adjustments^1,66^. Cells were grown in 96-well plates until 80% confluent, washed, and wells left with 50 µl control isotonic solution containing (in mM) NaCl 105, KCl 5, HEPES Acid 6, Na HEPES 4, NaHCO_3_ 5, mannitol 60, glucose 5, CaCl_2_ 1.3; pH 7.4. An additional 50 µl of isotonic control or experimental solution was added with drugs at twice the desired final concentration. Drugs added include poly(I:C), probenecid, NEM, GPN, rapamycin, bafilomycin and chloroquine. The 21-mer and 16-mer siRNA constructs shown in Table 1 were based on sequences published by Kleinman *et al*.^25^, while the “non-targeting” scrambled RNA was Silencer® Negative Control No. 2 siRNA (#AM4613, ThermoFisher). After exposure for the indicated time, 20 μl of luciferin/luciferase solution was added to each well. Luminescence was read 10× on Luminoscan (ThermoFisher) with 100 ms integration time per well, and converted to ATP concentration with a standard curve corrected for any effect of drugs on the assay. Levels from mouse astrocytes were measured 45 min after drug addition.

### Lactate dehydrogenase assay

LDH released into the extracellular solution was measured as an indicator of cell membrane integrity as described^3^. Briefly, cells in a 96-well plate were exposed to control or poly(I:C) solutions or lysed with Triton X-100 and the reaction mixture prepared according to manufacturer’s instructions (Cytotoxicity Detection Kit LDH, Roche Applied Science). Dye absorbance was measured at 490 nm and converted to LDH concentration using LDH standards.

### Measurement of lysosomal pH

Lysosomal pH was measured as described^63^. In brief cells were grown in black 96-well plates, rinsed and incubated with 5 µM LysoSensor Yellow/Blue DND 160 (Invitrogen Corp.) for 3 min followed by 15 min post-incubation. Lysosomal pH was determined from the ratio of light excited at 340 nm vs. 380 nm (>520 nM em) using a plate reader (Thermo Electron Corp.) and calibrated by exposing cells to 10 μM H^+^/Na^+^ ionophore monensin and 20 μM H^+^/K^+^ ionophore nigericin in (in mM) 20 MES, 110 KCl and 20 NaCl at pH 4.0–6.0 for 15 min.

### Measurement of cytoplasmic Ca^2+^

Cytoplasmic Ca^2+^ was measured as described^29^. In brief, ARPE-19 cells grown on 25 mm glass coverslips were loaded with 10 µM Fura-2 AM (acetoxymethyl ester, #F1221, ThermoFisher Scientific) with 0.02% pluronic F-127 (#P3000MP, ThermoFisher Scientific) for 45 min at 37 °C. Cells were then washed, mounted in a perfusion chamber and visualized using a 40 × objective on a Nikon Diaphot microscope (Nikon, Melville, NY) as described^67^. Ratiometric measurements were performed by alternating the excitation wavelength from 340 to 380 nm and quantifying emission ≥512 nm with a charge-coupled device camera (Photon Technologies International, Lawrenceville, NJ). Cells were perfused with isotonic solution with poly(I:C) ± apyrase washed over the cells. The *ImageJ Pseudocolor Look-Up Table* “16 colors” was used for displayed images, but not quantification^68^.

### Beta- hexosaminidase

ARPE-19 cells grown in 24-well plates were bathed with isotonic solution containing poly(I:C) (50 µg/ml) for 1 hr. A 50 µl sample of supernatant was incubated 1:1 with 1 mM *p*-nitrophenyl *N-acetyl β-D*-glucosaminide in 0.1 M of citrate buffer for 1 hr at 37 °C. The reaction was stopped by adding 200 µl of 0.4 M of glycine (pH = 10.7). For detection, absorbance was measure at 405 nm.

### Immunoblots of phosphorylated TLR3

Approaches are based upon published work^69^ with the following modifications. ARPE-19 cells were incubated either control or poly(I:C) (10 µg/ml) for 5 min and protein extracted. 15 µg protein was separated by SDS-PAGE and processed with a primary antibody to Phospho-TLR3 Tyr759 (1:200; ThermoFisher Scientific; PA5-23120). The membrane was stripped and reprobed with GAPDH antibody (1:10,000, Cell Signaling; mAb#2118). Blots were visualized using chemiluminescence with a horseradish peroxidase linked 2^nd^ antibody (1:5000; GE Healthcare).

### Acid phosphatase release

Acid phosphatase activity was quantified with colorimetric assay kit following manufacturer’s instructions (Sigma-Aldrich Corp, CS0740). Extracellular samples were mixed with substrate P-nitrophenyl phosphate (pNPP) and incubated for 30 min at 37 °C, pH = 4.8. The resulting p-Nitrophenol product was detected at 405 nm. Where possible, the same samples were used to measure both acid phosphatase and ATP.

### Microscopy

Cultured rat astrocytes were fixed with 4 °C methanol/acetone (80%/20%) for 20 min, then blocked with 10% goat serum in a phosphate buffered saline solution (PBS) for 90 min. Rabbit anti-glial fibrillary acidic protein (GFAP, 1:250 dilution, #MAB360, EMD Millipore, Temecula, CA) polyclonal antibody was added overnight at 4 °C, followed by a goat anti-rabbit IgG Alexa-Fluor 488 secondary antibody (1:500 dilution, Abcam Inc, Cambridge MA) for 60 min. For Lamp3 staining, ARPE-19 cells were fixed with 4% paraformaldehyde, washed, and incubated with Lamp3 (CD63 mouse monoclonal, 1:200 #MEM 259, Abcam) and then with anti-mouse secondary (1:500, Abcam). For VNUT localization, astrocytes were fixed in 4% formaldehyde for 15 min, rinsed with Dulbecco’s PBS and permeabilized at in a PBS + 0.1% Triton X-100 for 10 min, then blocked with 10% goat serum in SuperBlock blocking buffer (#37515, ThermoFisher Scientific) for 60 min. Anti-Vesicular Nucleotide Transporter (VNUT antibody) (1:1000 dilution, #ABN83, EMD Millipore, Temecula, CA) and Lamp3 (10 µg/ml concentration, #ab8219, Abcam, Cambridge, MA) primary antibodies were added overnight at 4 °C, followed by incubation of goat anti- Guinea Pig IgG Alexa-Fluor 568 (1:500 dilution) and anti-mouse IgG Alexa-Fluor 488 (1:500 dilution) secondary antibodies for 60 min. Slides were mounted in Slow Fade Gold Antifade Mountant and imaged using a Nikon fluorescent microscope (Eclipse E600). ARPE-19 cells were incubated with MANT-ATP (10 µM, ex. 360 nm, #M12417) and LysoTracker Red DND99 (50 nM, ex 564 nm L7528 both ThermoFisher Scientific) for 1 hr at 37 °C. RPE cells from LC3-GFP mice were imaged at 488 nm. Images were obtained in Nikon A1R confocal microscope system at the University of Pennsylvania Live Cell Imaging Core with NIS-Elements software or Nikon Eclipse E600 epifluorescent microscope (Nikon Inc.). Poly (I:C)-fluorescein (ThermoFisher Scientific) was added in medium at 10 µg/ml for indicated time. Cells were then incubated with 100 nM LysoTracker Red DND-99 for 5 min, fixed with 4% Paraformaldehyde, washed, labeled with Hoechst and viewed under a Nikon E600 fluorescent microscope. To monitor appearance of lysosomal lumen protein on the plasma membrane, cells were exposed to poly(I:C), ML-SA1 or control medium for 30 min with anti-rat monoclonal LAMP1–1DB4 (#ab25245, Abcam Inc.) at 37 °C for 30 min. Cells were washed, fixed in formaldehyde, then exposed to Alexa-488 conjugated anti–rat secondary antibodies (ThermoFisher Inc.) for 60 min at room temperature. After staining with DAPI, cells were visualized with confocal microscopy as above and processed with ImageJ^68^.

### Data Analysis

All data are expressed as mean ± standard error of the mean. Significance was defined as p < 0.05 and was determined using a one-way ANOVA followed by an appropriate post-Hoc test using SigmaStat software (Systat Software, Inc., San Jose, CA) unless otherwise noted. On occasions when data were not normally distributed, analysis was performed on ranks and the Mann-Whitney Rank Sum test was used. ATP/acid phosphatase levels were occasionally normalized to the mean value for each plate or set to account for trial by trial variation in absolute numbers.

Portions of this work have previously appeared in abstract form^70,71^.

### Data availability statement

All data generated or analyzed during this study are included in this published article.

### Summary Statement

This study identifies crosstalk between the innate immune system, autophagy and purinergic signaling. Lysosomal exocytosis of ATP is triggered by multiple agonists to TLR3 including “non-targeting” scrambled siRNA and involves TRPML1.

## Electronic supplementary material

Supplementary Figure 1
