# Supplementary material for: Stimulation of TLR3 triggers release of lysosomal ATP in astrocytes and epithelial cells that requires TRPML1 channels
Source: Sci Rep. 2018 Apr 10;8:5726. doi: 10.1038/s41598-018-23877-3 (PMC5893592; doi:10.1038/s41598-018-23877-3)
Supplement: Supplementary file 1 — Supplementary Figure 1 [file 41598_2018_23877_MOESM1_ESM.pdf]

Title: Stimulation of TLR3 triggers release of lysosomal ATP in astrocytes and epithelial cells that requires TRPML1 channels

Authors: Jonathan M. Beckel, Néstor Más Gómez, Wennan Lu, Keith E. Campagno, Bardia Nabet, Farraj Albalawi, Jason C. Lim, Kathleen Boesze-Battaglia, and Claire H. Mitchel.

Supplemental Figure 1

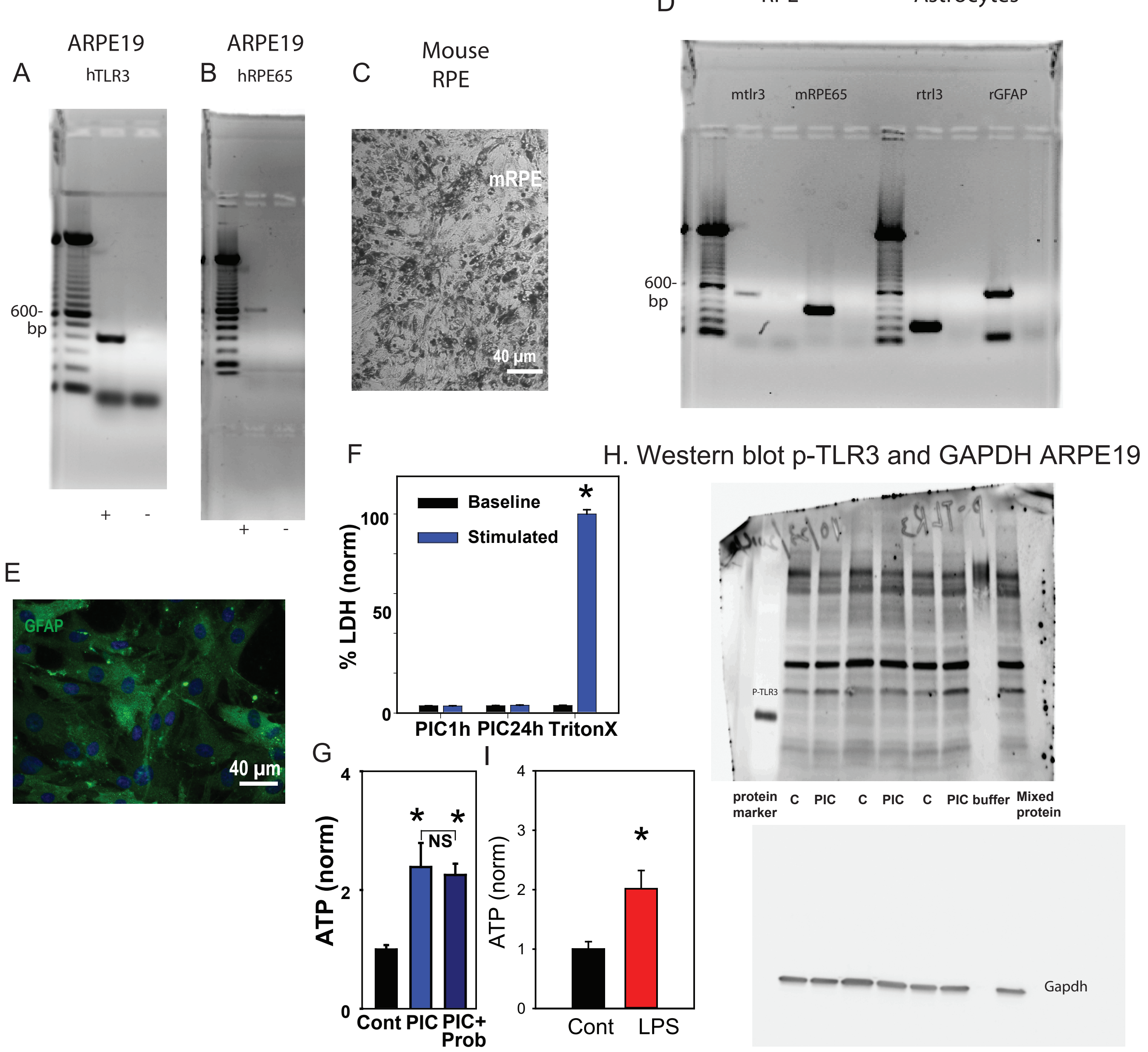

A. PCR gel for human TLR3 in ARPE19 cells. Marker bars are 100 bp. + with, - without reverse transcriptase.
B. PCR gel for human RPE65 in ARPE19 cells.
C. Mouse RPE cells in culture, displaying pigment.
D. PCR gel for mouse TLR3 and RPE65 in mouse RPE cells, and rat TLR3 and GFAP in rat astrocytes.
E. Cultured rat optic nerve head astrocytes stained with GFAP (green) and DAPI (Blue).
F. Poly(I:C) (10µg/ml) did not elevate lactose dehydrogenase (LDH) levels in astrocytes after 1 or 24 hrs but lysing cells with Triton X did, n=4
G. ATP release by poly(I:C) was not blocked by 1 mM probenecid (n=30)
H. Western blots showing phospho-TLR3 in ARPE19 cells exposed to poly(I:C) (top gel) and reblotted for GAPDH (bottom).
I. Addition of LPS (0111:B4) at 1 µg/ml for 1 hr also increased the release of ATP from ARPE19 cells.
